# Impedance Model of Cylindrical Nanowires for Metamaterial Applications

**DOI:** 10.3390/nano9081104

**Published:** 2019-08-01

**Authors:** Mehboob Alam, Ali Mahmood, Shahida Azam, Madiha Saher Butt, Anwar Ul Haq, Yehia Massoud

**Affiliations:** 1 Department of Electrical Engineering, Mirpur University of Science and Technology (MUST), Mirpur-10250 (AJK), Pakistan; 2 Department of Informatics (I-13), Bolzmanstr. 3, Technical University Munich, 85748 Garching, Germany; 3 School of Systems and Enterprises, Stevens Institute of Technology, Hoboken, NJ 07030, USA

**Keywords:** nanophotonics, metamaterial, nanowire, Mie solution, impedance, plasmonics, nanocylinder, nanomaterial

## Abstract

In metamaterials, metallic nanowires are used for creating artificial materials to functionalize them for various nanophotonics applications. Strong polarization-dependent response coupled with complex dielectric function at optical frequencies gives additional degrees of freedom to achieve scattering, absorption, and other benefits that go much beyond what is possible with conventional materials. In this paper, we propose an extended cylindrical wave impedance approach at optical frequencies to model the internal and external impedance of the metallic nanowire. Equivalent analytical expression for the scattering, extinction, and absorption cross-sectional area efficiencies are derived in terms of impedances. The motivation is to develop an all-mode solution (${\mathrm{TM}}_{n}$ and ${\mathrm{TE}}_{n}$ modes), by bringing the complex problem of plasmonic nanowire to linear system theory, where established methods can be applied to enable new applications. The equivalence of the impedance solution is compared with electromagnetic field solution and numerical full-wave field simulations. The proposed solution is accurate and may contribute to the rapid and efficient future designs for the metallic nanowire-based nanophotonic metamaterials.

## 1. Introduction

In the past decade, there has been growing interest in the rapidly evolving field of metamaterial and its exciting applications [1,2,3,4,5]. The essentially interesting behavior is due to the existence of artificial material with both negative permittivity and permeability [6,7,8]. Among these structures, the basic building blocks of artificial materials often consists of metallic elements, which are repeated periodically to achieve the desired metamaterial properties [9,10,11]. The advantage of using plasmonic structure is two-fold. Firstly, at optical frequency they exhibit negative permittivity. Secondly, metal as a constituent structure in a metamaterial can promise reduction in plasma frequency, thereby allowing negative permittivity to be achieved at much lower frequencies [12,13]. The idea of using metallic structures to dilute plasma frequency was first coined by J. B. Pendry et al. [7]. In these metallic structures, nanowires form the basic unit cell and are periodically assembled to form a cubic lattice. The built structures are analytically proven using laws of physics to reduce plasma frequency, and was later experimentally verified at microwave frequencies [6].

Metallic nanowires are basic building blocks of rapidly evolving field of artificial metamaterials and their shape dependent optical response can be used to render macroscopic object invisible [14]. Cylindrical nanowires are also used to develop epsilon-near-zero (ENZ) metamaterials [15] and can combine with other metallic nanostructures to develop near-zero index of refraction ($\eta =\sqrt{\epsilon \mu }\approx 0$) meta-devices and metamaterials. The developing field of near-zero index metamaterials finds applications in waveguides, quantum information processing, optical isolators, sub-wavelength imaging and hyperlensing [16].

In modeling plasmonics nanostructures, there are two approaches to represent the resonant structures using circuit elements. The first method is the quasi-static voltage and current model, which uses derived values of voltages and currents for the dominant mode (dipole mode) and was only applied to model metallic nanoparticle at optical frequencies [17,18]. The closed form derivation of nanowire using quasi-static voltage and current model (resonant model) is not possible as neither the dipole mode is dominant nor the integration of field is possible, even after ignoring the end effects (energy scattered by the ends) [19]. The second method is the wave impedance approach, where the ratio of electric to magnetic fields (E/H) defines the equivalent impedance [20]. The wave impedance approach is only applied to metallic nanoparticle and is the closest approximation of the Maxwell equations, which also models the multimode excitation of metallic nanoparticles [20,21].

A self-inductance formulation of the metallic wires at microwave frequencies by increasing the number of inductance elements was introduced later, which further lowered the plasma frequency by increasing effective mass [22]. However, a self-inductance-based formulation of current-carrying conductor is a derivation under quasi-static approximation, where the size of the structure is much smaller than wavelength of the incident wave. Please note that in all these contributions, the basic building block is a metallic thin wire, forming periodic array of resonant element, thus giving desired electromagnetic properties of artificial permittivity [7]. The lumped impedance representation is a useful concept for characterizing resonant structures in metamaterials. In circuit theory, it is the exchange of energy from inductor to capacitor within a circuit, which simplifies the complex behavior of plasmon resonance [17,18,20,23,24,25]. The network build with circuit components can use systematic nodal and mesh analysis to develop application specific solutions. The main contributions of this work are as follows:

- New cylindrical wave impedance approach (impedance defined as E/H) is used at optical frequency, which uses cylindrical harmonic functions to derive expression for the internal and external impedances of the metallic nanowire.
- Equivalent analytical expression for the scattering ($Q_{sca}$) and extinction ($Q_{ext}$) cross-sectional area efficiencies of the nanowire (basic unit cell in metamaterial) is derived in terms of impedances.
- Derived expressions are in terms of length, radius, and dielectric function of the nanowires, thus preserving macroscopic plasmon properties of the metal at optical frequency.

Considering the strong polarization-dependent response of the nanowire, we included the derivation of both ${\mathrm{TM}}_{n}$ and ${\mathrm{TE}}_{n}$ mode in the next section, with integer values of *n* (varying from 0 to ∞) highlighting the excited mode. In next the section, we will briefly discuss exact field solution to the scattering, absorption, and extinction by a cylindrical nanowire. In Section 3, we present the proposed impedance derivation of a metallic cylindrical nanowire. Simulation and comparison with full-wave field solver is presented in Section 4. The paper is finally concluded in Section 5.

## 2. EM Field Solution to the Scattering, Absorption and Extinction in Circular Cylindrical Nanowire

**Figure 1:**
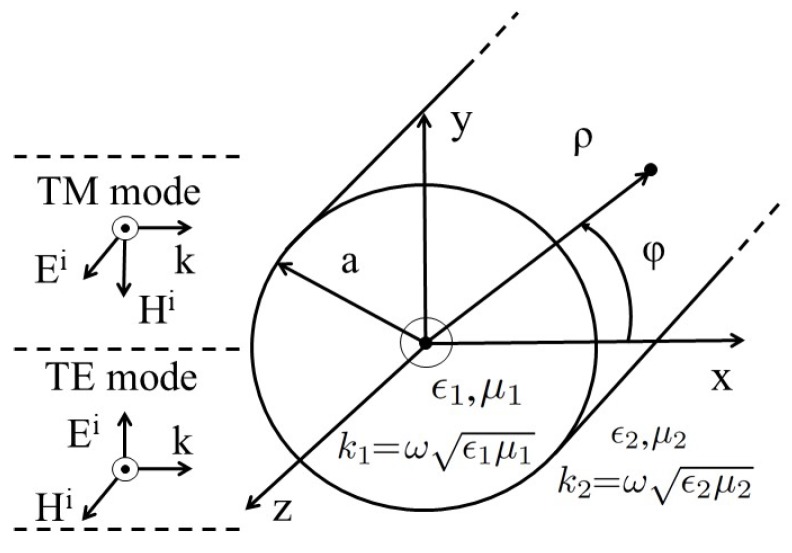
A uniform TM/TE plane wave with +x as direction of propagation in free space is incident on cylindrical nanowire of radius *a*.

Cylindrical harmonic functions appear in the class of problems that exhibit cylindrical symmetry. The field solution using Maxwell equations for scattering and absorption from a circular cylinder is exactly analogous to the Mie solution for the spherical geometries [26]. The solution can be achieved by considering either excitation of TM or TE mode. Considering *x* axis, as the direction of propagation, the two simple combinations i.e., with E field and H field along *y* and *z* axis or *z* and −y axis will lead to the generation of TE and TM modes respectively. Let us first consider a uniform TM plane wave excitation of circular cylinder with radius *a* oriented along the *z* axis, as shown in Figure 1. The expression for incident, scattering, and absorbed wave of the electric fields can be written as

(1) $$\begin{array}{cc} E^{in} & =\hat{a_{z}}E_{0}\sum_{n=-\infty }^{+\infty }j^{-n}J_{n}\left(k_{2}\rho \right)e^{jn\phi },\;\;\rho \geq a \end{array}$$

(2) $$\begin{array}{cc} E^{sca} & =\hat{a_{z}}E_{0}\sum_{n=-\infty }^{+\infty }a_{n}H_{n}^{\left(2\right)}\left(k_{2}\rho \right)e^{jn\phi },\;\;\rho \geq a \end{array}$$

(3) $$\begin{array}{cc} E^{abs} & =\hat{a_{z}}E_{0}\sum_{n=-\infty }^{+\infty }b_{n}J_{n}\left(k_{1}\rho \right)e^{jn\phi },\;\;a\geq \rho \geq 0 \end{array}$$

where $k_{1}=\omega \sqrt{\epsilon_{1}\mu_{1}}$, $k_{2}=\omega \sqrt{\epsilon_{2}\mu_{2}}$, with permitivities ($\epsilon_{1},\;\epsilon_{2}$) and permeabilities ($\mu_{1},\;\mu_{2}$) for the two regions defined in Figure 1. The other functions are $J_{n}$ (Bessel function of first kind) and $H_{n}^{\left(2\right)}$ (Hankel function of second kind). Applying the boundary conditions (at ρ=a), we can solve for the coefficients $a_{n}$ and $b_{n}$, and their final value can be written as

(4) $$a_{n}=\frac{mJ_{n}\left(mx\right)J_{n}^{\prime }\left(x\right)-J_{n}\left(x\right)J_{n}^{\prime }\left(mx\right)}{J_{n}^{\prime }\left(mx\right)H_{n}^{\left(2\right)}\left(a\right)-mJ_{n}\left(mx\right)H_{n}^{(2)\prime }\left(x\right)},$$

(5) $$b_{n}=\frac{J_{n}\left(mx\right)J_{n}^{\prime }\left(x\right)-mJ_{n}^{\prime }\left(mx\right)J_{n}\left(x\right)}{J_{n}\left(mx\right)H_{n}^{(2)\prime }\left(x\right)-mJ_{n}^{\prime }\left(mx\right)H_{n}^{\left(2\right)}\left(x\right)},$$

where the variables $x=k_{2}a$ and $m=k_{1}/k_{2}$ and the prime shows differentiation with respect to the arguments. We take special case of TM with E parallel to cylinder axis. The details can be found in [27,28,29] and in other literature. As a result, of this simplification for the TM mode, $a_{n}$ vanishes and only $b_{n}$ exist with finally derived expression for $Q_{sca}$ and $Q_{ext}$ written as

(6) $$Q_{sca}^{TM}=\frac{W_{sca}^{TM}}{2aLI_{i}}=\frac{2}{x}\left[|b_{0}{|}^{2}+2\sum_{n=1}^{+\infty }{\left|b_{n}\right|}^{2}\right],$$

(7) $$Q_{ext}^{TM}=\frac{W_{ext}^{TM}}{2aLI_{i}}=\frac{2}{x}Re\left\{b_{0}+2\sum_{n=1}^{+\infty }b_{n}\right\},$$

where *L* is the length of cylinder and $I_{i}$ is the incident irradiance. Similarly, $W_{sca}^{TM}$ and $W_{ext}^{TM}$ is the rate at which energy is scattered or extincted, respectively. A similar analysis can be done for the excitation of the TE mode, when the incident H is along *z*-axis, as shown in Figure 1. However, we will only include the final results of $Q_{sca}$ and $Q_{ext}$ here, which can be written as

(8) $$Q_{sca}^{TE}=\frac{W_{sca}^{TE}}{2aLI_{i}}=\frac{2}{x}\left[{\left|a_{0}\right|}^{2}+2\sum_{n=1}^{+\infty }{\left|a_{n}\right|}^{2}\right],$$

(9) $$Q_{ext}^{TE}=\frac{W_{ext}^{TE}}{2aLI_{i}}=\frac{2}{x}Re\left\{a_{0}+2\sum_{n=1}^{+\infty }a_{n}\right\}.$$

Please note that the absorption cross-sectional area efficiency in both cases is the difference of $Q_{ext}$ and $Q_{sca}$, and can be written as

(10) $$Q_{abs}^{TM/TE}=Q_{ext}^{TM/TE}-Q_{sca}^{TM/TE}.$$

**Figure 2:**
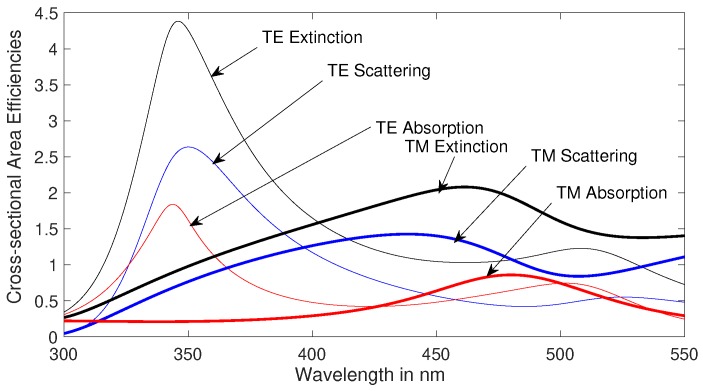
Extinction, scattering and absorption cross-sectional efficiencies (dimensionless) plotted using field solution for the excited TE and TM modes for a 30 nm (nanometer) radius gold nanowire.

In Figure 2, we plot extinction, scattering and absorption cross-sectional efficiencies using full-wave field solver for the excited TE and TM modes for a 30nm radius gold cylindrical nanowires using modified Drude dielectric function [30]. The surface plasmon resonance phenomena of extinction, scattering, and absorption cross-sectional areas shown in Figure 2 can also be explained with the help of electrical circuit analysis. In circuit theory, it is the exchange of energy from inductor to capacitor within a circuit, which simplifies the complex behavior of plasmon resonance. In the quasi-static approximation (radius ≪λ), the metallic nanowire internal and external impedance will be primarily inductive and capacitive respectively, with equivalent circuit analogous to parallel resonance circuit. Therefore, at resonance the displacement surface current of nanowire will be in-phase with the excitation as the imaginary components of internal and external impedance cancel out each other. Please note that the impedance, just like parallel resonance circuit, changes with wavelength, with equivalent circuit being capacitive and inductive before and after the plasmon resonance peak, respectively.

The field solution, in wave analysis is often known as the fundamental solution to any electromagnetic problem. The derived values in this section will be used in subsequent sections to support our proposed impedance model.

## 3. Proposed Wave Impedance Model of Metallic Cylindrical Nanowires

In this section, we will discuss the derivation of the proposed impedance model for the excited modes in metallic cylindrical nanowire.

### 3.1. **TM** Mode: Wave Impedance and Cross-Sectional Areas Efficiencies (Scattering, Absorption and Extinction)

Let us consider a uniform plane wave propagating along +x, with **E** and **H** along +z and −y directions, respectively. This will excite TM mode in the nanowire. To start with the derivation of impedance, all we need is the relationship of $E_{z}^{sca}$ (2) and $E_{z}^{abs}$ (3) and we can drive expressions of $H_{\phi }^{sca}$ and $H_{\phi }^{abs}$ using Maxwell’s equation as

(11) $$\begin{array}{cc} H_{\phi }^{sca} & =\frac{E_{0}k_{2}}{j\omega \mu_{0}}\sum_{n=-\infty }^{+\infty }a_{n}H_{n}^{(2)\prime }\left(k_{2}\rho \right)e^{jn\phi }, \end{array}$$

(12) $$\begin{array}{cc} H_{\phi }^{abs} & =\frac{E_{0}k_{1}}{j\omega \mu_{1}}\sum_{n=-\infty }^{+\infty }b_{n}J_{n}^{\prime }\left(k_{1}\rho \right)e^{jn\phi }. \end{array}$$

The details of electric and magnetic fields used in these derivations can be found in [27,28,29] and in other literature. Let us use (2), (3), (11) and (12) to derive expressions for the internal and external impedances.

#### 3.1.1. Internal Impedance

It is worth correlating the approach adopted here in our proposed model with the wave impedance in free space using rectangular coordinates. The impedance relates E and H field with the following relationship

(13) $$Z=\frac{E}{H}=\sqrt{\frac{\mu_{0}}{\epsilon_{0}}}=\eta .$$

where $\mu_{0}$ and $\epsilon_{0}$ are the permeability and permittivity of free space. The formulation works well at microwave for various mode of transmission. Here, we propose that for geometrically well-conditioned problem, a similar approach can be extended up to optical frequencies and the derived impedances can be used to adequately describe the behavior of metal at optical frequencies. In case of TM mode, with *n* varying from 0 to ∞, the internal impedance (at ${lim}_{\rho \to a}$) of the metallic nanowire, using the values of $E_{z}^{abs}$ (3) and $H_{\phi }^{abs}$ (12) can be formulated as

(14) $$Z_{int}^{{TM}_{n}}{|}_{lim_{\rho \to a}}=-\frac{V_{int}^{TM}\left(k_{1}a\right)}{I_{int}^{TM}\left(k_{1}a\right)}=-\frac{E_{z}^{abs}}{H_{\phi }^{abs}}=-\frac{j\omega \mu_{1}J_{n}\left(k_{1}a\right)}{k_{1}J_{n}^{\prime }\left(k_{1}a\right)},$$

where $Z_{int}^{{TM}_{n}}$ is the internal impedance of the excited ${\mathrm{TM}}_{n}$ mode. Please note that the -ve sign is due to inward waves, which in our case are contributing to absorption. Let us now express $Z_{int}^{TM}$, which is an impedance rational function consisting of Bessel function of first kind to a continued fraction. A further simplification of (14), the rational function consisting of $J_{n}\left(k_{1}a\right)$ and $J_{n}^{\prime }\left(k_{1}a\right)$ gives us following continued fraction

(15) $$Z_{int}^{TM}=\eta_{1}\times \frac{1}{\frac{n}{jk_{1}a}+\frac{1}{\frac{\left(2n-1\right)}{jk_{1}a}+\frac{1}{\frac{\left(2n-3\right)}{jk_{1}a}+...+\eta_{1}}}}$$

**Figure 3:**
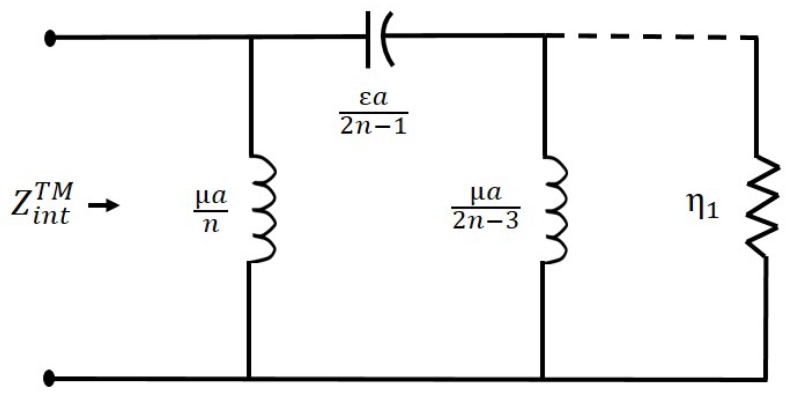
Representation of internal impedance of the excited ${\mathrm{TM}}_{n}$ mode in the cylindrical nanowire forming a ladder network.

The $Z_{int}^{TM}$ (for the excited ${\mathrm{TM}}_{n}$) expressed as ladder network with various values of capacitors and inductors is shown in Figure 3.

#### 3.1.2. External Impedance

External impedance of metallic nanowire will be derived in a similar fashion. The difference is in the formulation, which is now governed by the values of $E_{z}^{sca}$ (2) and $H_{\phi }^{sca}$ (11) in the medium $k_{2}$, and is written as

(16) $$Z_{ext}^{{TM}_{n}}{|}_{lim_{\rho \to a}}=\frac{V_{ext}^{TM}\left(k_{2}a\right)}{I_{ext}^{TM}\left(k_{2}a\right)}=\frac{E_{z}^{sca}}{H_{\phi }^{sca}}=\frac{j\omega \mu_{2}H_{n}^{\left(2\right)}\left(k_{2}a\right)}{k_{2}H_{n}^{(2)\prime }\left(k_{2}a\right)},$$

where $Z_{ext}^{{TM}_{n}}$ is the external impedance of the excited *n*th TM mode. A further derivation of impedance in to ladder network and equivalent circuit is discussed in simulation and results section. In the next section, we will drive expressions for various cross-sectional area efficiencies using these proposed internal and external wave impedance values. Please note that the previous known expressions for the efficiencies are derived using electromagnetic field (E and H) analysis.

#### 3.1.3. **TM** Mode: Cross-Sectional Areas Efficiencies Using Impedances

In general, the derivation of the impedance in the absence of frequency dependent medium should be straight forward. However, we observe that the presence of metallic nanowire with large frequency dependent negative real part of its dielectric function makes it a non-trivial solution. In this section, we will use $Z_{int}^{{TM}_{n}}$ and $Z_{ext}^{{TM}_{n}}$ derived in the preceding sections to carefully derive expression for the impedance-based $Q_{sca}$, $Q_{ext}$ and $Q_{abs}$.

**Figure 4:**
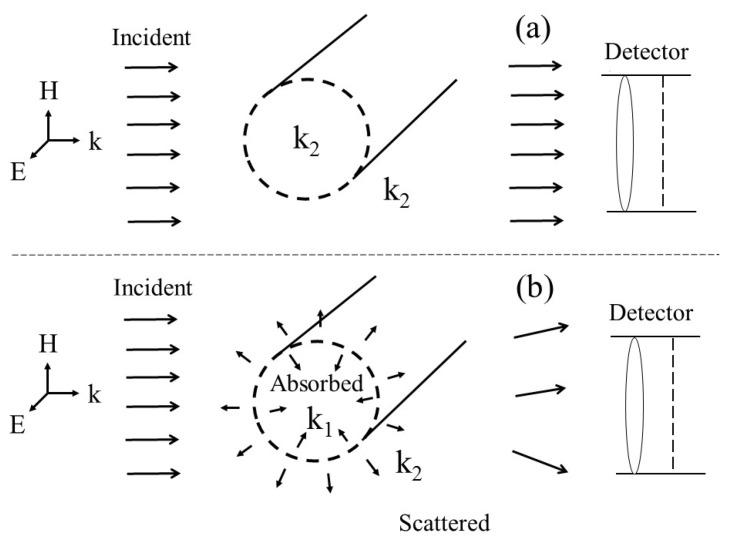
Extinction of a single nanowire. (**a**) The internal medium property $k_{2}=\omega \sqrt{\epsilon_{2}\mu_{2}}$ is same as external medium property $k_{2}$. (**b**) The material property $k_{1}=\omega \sqrt{\epsilon_{1}\mu_{1}}$ (of cylinder) is not the same as $k_{2}$ (external medium).

In case of extinction, it is the ratio of the removal of energy from the incident wave due to absorption and scattering. We will consider two different cases of excitation of a nanowire for the derivation of our proposed model, as shown in Figure 4a,b. The first is with the metallic cylindrical nanowire dielectric function different from the embedding medium and second is with dielectric function same as the embedding medium. The dotted nanowire given in Figure 4a shows the presence of an imaginary particle of radius *a*, with a material property of $k_{2}=\omega \sqrt{\epsilon_{2}\mu_{2}}$, which is the same as of the external medium. Therefore, the incident waves will not be effected by the nanowire. A metallic nanowire with the material property of $k_{1}$, different from the surrounding medium is shown in Figure 4b. Hence, the incident energy will be disrupted, resulting in scattered and absorbed waves, as shown in the Figure 4b.

In both cases, the excitation of surface charges due to incident field at ρ=a of nanowire produces surface currents. For the development of solution, the values of surface current are defined as:

- $I_{ext}^{TM}\left(k_{1}a\right)$: Surface current due to nanowire with property $k_{1}$, supporting scattering waves.
- $I_{ext}^{TM}\left(k_{2}a\right)$: Surface current due to nanowire with property $k_{2}$, supporting scattering waves.
- $I_{int}^{TM}\left(k_{1}a\right)$: Surface current due to nanowire with property $k_{1}$, supporting waves absorption.
- $I_{int}^{TM}\left(k_{2}a\right)$: Surface current due to nanowire with property $k_{2}$, supporting waves absorption.
- $Z_{\beta }$: The ratio between $I_{int}^{TM}\left(k_{2}a\right)$ and $I_{ext}^{TM}\left(k_{2}a\right)$.

**Figure 5:**
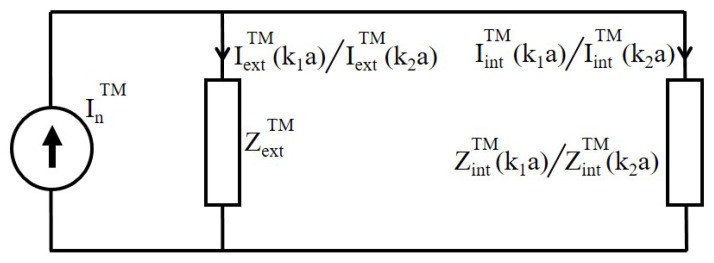
For the excitation of TM mode, a current generator $I_{n}^{TM}$ at the boundary of cylindrical nanowire, get distributed as internal ($I_{int}^{TM}\left(k_{1}a\right)$/$I_{int}^{TM}\left(k_{2}a\right)$) and external ($I_{ext}^{TM}\left(k_{1}a\right)$/$I_{ext}^{TM}\left(k_{2}a\right)$) current based on properties ($k_{1}$/$k_{2}$) of the nanowire, as shown in Figure 4a,b. Whereas $Z_{int}^{TM}\left(k_{1}a\right)$/$Z_{int}^{TM}\left(k_{2}a\right)$ represents internal impedance of the nanocylinder with property $k_{1}$/$k_{2}$ and $Z_{ext}^{TM}\left(k_{2}a\right)$ is the external impedance of the nanowire.

The defined currents are further explained in Figure 5. The figure represents two different scenarios. Firstly, we consider a metallic cylindrical nanowire with properties ($k_{1}=\omega \sqrt{\epsilon_{1}\mu_{1}}$), different then the surrounding medium ($k_{2}=\omega \sqrt{\epsilon_{2}\mu_{2}}$) and leads to different internal ($I_{int}^{TM}\left(k_{1}a\right)$) and external ($I_{ext}^{TM}\left(k_{1}a\right)$) surface currents and impedances. In second case, the material properties of nanowire and medium are same ($k_{2}=\omega \sqrt{\epsilon_{2}\mu_{2}}$) with $I_{int}^{TM}\left(k_{2}a\right)$ and $I_{ext}^{TM}\left(k_{2}a\right)$ as internal and external surface currents. Please note that in both cases, embedding medium is same with property $k_{2}$ and leads to same $Z_{ext}^{TM}$ as external impedance. At the boundary of metallic cylindrical nanowire, the current source ($I_{n}^{TM}$) becomes a generator responsible for the excitation of surface currents responsible for generating various modes, as shown in Figure 5. The $I_{ext}^{TM}\left(k_{1}a\right)$ and $I_{ext}^{TM}\left(k_{2}a\right)$ are the current due to nanowire with material properties $k_{1}$ and $k_{2}$ respectively and are also explained in Figure 4 and Figure 5. The value of $I_{ext}^{TM}\left(k_{1}a\right)$ and $I_{ext}^{TM}\left(k_{2}a\right)$ for the two cases can be written as

(17) $$I_{ext}^{TM}\left(k_{1}a\right)=\frac{I_{n}^{TM}Z_{int}^{TM}\left(k_{1}a\right)}{Z_{int}^{TM}\left(k_{1}a\right)+Z_{ext}^{TM}\left(k_{2}a\right)},$$

(18) $$I_{ext}^{TM}\left(k_{2}a\right)=\frac{I_{n}^{TM}Z_{int}^{TM}\left(k_{2}a\right)}{Z_{int}^{TM}\left(k_{2}a\right)+Z_{ext}^{TM}\left(k_{2}a\right)}.$$

Let us also define value of gain factor $Z_{\beta }$ using (14) and (16) as the ratio between the two current as

(19) $$Z_{\beta }=\frac{I_{int}^{TM}\left(k_{2}a\right)}{I_{ext}^{TM}\left(k_{2}a\right)}=\frac{J_{n}^{\prime }\left(k_{2}a\right)}{H_{n}^{(2)\prime }\left(k_{2}a\right)}.$$

The scattering cross-section is defined as ratio of total scattered power ($W_{s}^{TM}$) to the power density of the incident wave (*P*). We can define the scattered power due to excited mode (TM) as

(20) $$W_{s}^{TM}=2\pi aL\frac{{\left(I_{s}^{TM}\right)}^{2}\eta_{2}}{2},$$

where the embedding medium have impedance of $\eta_{2}$, which is equal to $\sqrt{\mu_{2}/\epsilon_{2}}$. In calculating scattering power, we ignore the end effects (energy scattered by the ends, as L>>a) and consider only the energy scattered per unit length by constructing an imaginary surface of length *L* and radius *a*, resulting in 2πaL contribution in (20). Please note that the analytical solution (electromagnetic field solution) also accounts for similar approximation, where the solution only exist for infinitely long cylinder or the length (*L*) of cylinder is much larger than the radius (*a*) [19]. In (20), the $I_{s}^{TM}$ is the current, which supports excitation of scattering waves in TM mode. It is defined using $b_{n}^{TM}$ (scattering gain factor) and the incident current wave generator ($I_{n}^{TM}$) as

(21) $$I_{s}^{TM}=I_{n}^{TM}b_{n}^{TM},$$

where the amplitude of cylindrical wave generator is given as

(22) $$I_{n}^{TM}=E_{0}\frac{\sqrt{\frac{2}{\pi k\rho }}}{\eta_{2}}=H_{0}\sqrt{\frac{2}{\pi k\rho }},$$

where ρ is the distance from z-axis. We have taken asymptotic expression of the wave ($H_{n}\left(k\rho \right)=\sqrt{2/(\pi k\rho )}$) for the cylindrical wave generator [19]. Please note that Hankel functions $\left(H_{n}\left(k\rho \right)\right)$ are used to represent amplitude of traveling cylindrical waves [27] and in this case represent maximum amplitude of cylindrical wave generator. The gain factor $b_{n}^{TM}$ in (21) is the fraction of difference in surface current due to existence of nanowire with material properties $k_{1}$ and can be written as

(23) $$b_{n}^{TM}=1-\frac{I_{ext}^{TM}\left(k_{1}a\right)}{I_{ext}^{TM}\left(k_{2}a\right)}=\frac{I_{ext}^{TM}\left(k_{2}a\right)-I_{ext}^{TM}\left(k_{1}a\right)}{I_{ext}^{TM}\left(k_{2}a\right)},$$

Using values of $I_{ext}^{TM}\left(k_{1}a\right)$ and $I_{ext}^{TM}\left(k_{2}a\right)$ from (17) and (18), the gain factor $b_{n}^{TM}$ can be written as

(24) $$b_{n}^{TM}=\frac{Z_{\beta }\left[Z_{int}^{TM}\left(k_{1}a\right)-Z_{int}^{TM}\left(k_{2}a\right)\right]}{\left[Z_{int}^{TM}\left(k_{1}a\right)+Z_{ext}^{TM}\left(k_{2}a\right)\right]}.$$

Let us know prove that this gain factor ($b_{n}^{TM}$) is in-fact field solution’s co-efficient of expansion. Please note that the gain defined by (24), exist due to presence of metallic nanowire with property $k_{1}=\omega \sqrt{\epsilon_{1}\mu_{1}}$ to support TM mode. Using (19), (21) and (24) in (20), the total scattered power (0<n<∞) for the TM modes can be written as

(25) $$\begin{array}{c} W_{s}^{TM}=\frac{2aLE_{0}^{2}}{\eta_{2}kr}\sum_{n=0}^{\infty }{\left|\frac{Z_{\beta }\left[Z_{int}^{TM}\left(k_{1}a\right)-Z_{int}^{TM}\left(k_{2}a\right)\right]}{\left[Z_{int}^{TM}\left(k_{1}a\right)+Z_{ext}^{TM}\left(k_{2}a\right)\right]}\right|}^{2}, \end{array}$$

(26) $$W_{s}^{TM}=\frac{2aLE_{0}^{2}}{\eta_{2}kr}\sum_{n=0}^{\infty }{\left|b_{n}^{TM}\right|}^{2}.$$

Hence, the solution for the TM mode for the scattering cross-sectional area efficiencies have a general form, which can be written as

(27) $$Q_{sca}=\frac{W_{s}^{TM}}{2aLP}=\frac{2}{x}\sum_{n=0}^{\infty }{\left|\frac{Z_{\beta }\left[Z_{int}^{TM}\left(k_{1}a\right)-Z_{int}^{TM}\left(k_{2}a\right)\right]}{\left[Z_{int}^{TM}\left(k_{1}a\right)+Z_{ext}^{TM}\left(k_{2}a\right)\right]}\right|}^{2},$$

where *P* is given as

(28) $$P=\frac{E_{0}^{2}}{2\eta_{2}}.$$

Using (14) and (16) in (27), the absolute term can be further reduced as

(29) $$\begin{array}{c} \left.\begin{array}{c} \frac{Z_{\beta }\left[Z_{int}^{TM}\left(k_{1}a\right)-Z_{int}^{TM}\left(k_{2}a\right)\right]}{\left[Z_{int}^{TM}\left(k_{1}a\right)+Z_{ext}^{TM}\left(k_{2}a\right)\right]}=\\ \frac{J_{n}\left(k_{1}a\right)J_{n}^{\prime }\left(k_{2}a\right)-mJ_{n}^{\prime }\left(k_{1}a\right)J_{n}\left(k_{2}a\right)}{J_{n}\left(k_{1}a\right)H_{n}^{(2)\prime }\left(k_{2}a\right)-mJ_{n}^{\prime }\left(k_{1}a\right)H_{n}^{\left(2\right)}\left(k_{2}a\right)}=b_{n}. \end{array}\right. \end{array}$$

Please note that the initial assumption is of the plane wave propagating along +x, with **E** along +z axis. Therefore, from definition of (5) and (6), the scattering cross-sectional area efficiencies using (29) can be written as

(30) $$Q_{sca}=\frac{W_{s}}{2aLP}=\frac{2}{x}\sum_{n=0}^{\infty }\left[|b_{0}{|}^{2}+2\sum_{n=1}^{+\infty }{\left|b_{n}\right|}^{2}\right].$$

Please note that it is the same scattering cross-sectional area efficiency expression derived using field solution [29]. Similarly, an expression can be derived for extinction cross-sectional area efficiency by using our proposed wave impedance model. In metallic nanowire, an extinction area efficiency is expressed in terms of $Q_{ext}$. Please note that the only difference in derivation compared to the $Q_{sca}$ is that we take the real part of the gain factors rather than the absolute value. Therefore, extinction power of a metallic nanowire for the TM modes can be written as

(31) $$\begin{array}{c} W_{e}^{TM}=\frac{2aLE_{0}^{2}}{\eta_{2}kr}\sum_{n=0}^{\infty }Re\left\{\frac{Z_{\beta }\left[Z_{int}^{TM}\left(k_{1}a\right)-Z_{int}^{TM}\left(k_{2}a\right)\right]}{\left[Z_{int}^{TM}\left(k_{1}a\right)+Z_{ext}^{TM}\left(k_{2}a\right)\right]}\right\}, \end{array}$$

(32) $$W_{e}^{TM}=\frac{2aLE_{0}^{2}}{\eta_{2}kr}\sum_{n=0}^{\infty }Re\left\{b_{n}^{TM}\right\}.$$

The $Q_{ext}$ can then be generalized as

(33) $$Q_{ext}=\frac{W_{e}}{2aLP}=\frac{2}{x}\sum_{n=0}^{\infty }Re\left\{|b_{0}{|}^{2}+2\sum_{n=1}^{+\infty }{\left|b_{n}\right|}^{2}\right\},$$

which is the same extinction cross-sectional area efficiency expression derived using field solution [29]. We will now use (30), (33) and the relationship between cross-sectional areas efficiencies to write absorption cross-sectional area efficiency as

(34) $$Q_{abs}=Q_{ext}-Q_{sca}.$$

Please note that the equations of $Q_{sca}$ (30), $Q_{ext}$ (33) and $Q_{abs}$ (34) are expressed in terms of circuit elements and impedances using our proposed wave impedance approach. The impedances and their behavior are well known to us and can form basis for analysis and design of metallic nanowire-based nanophotonic applications.

### 3.2. **TE** Mode: Wave Impedance and Cross-Sectional Area Efficiencies (Scattering, Absorption and Extinction)

Let us consider uniform plane wave propagating along +x, with **E** and **H** along +y and +z directions, respectively. To start with the derivation of impedance, all we need is the relationship of $H_{z}^{sca}$, $H_{z}^{abs}$, $E_{\phi }^{sca}$ and $E_{\phi }^{abs}$, which are the TE components of the scattered and absorbed waves of the electric and magnetic fields respectively. From the preceding sections, the respective H and E fields values can be written as follows

(35) $$\begin{array}{cc} H_{z}^{sca} & =H_{0}\sum_{n=-\infty }^{+\infty }a_{n}H_{n}^{\left(2\right)}\left(k_{2}\rho \right)e^{jn\phi }, \end{array}$$

(36) $$\begin{array}{cc} H_{z}^{abs} & =H_{0}\sum_{n=-\infty }^{+\infty }b_{n}J_{n}\left(k_{1}\rho \right)e^{jn\phi }, \end{array}$$

(37) $$\begin{array}{cc} E_{\phi }^{sca} & =-\frac{H_{0}k_{2}}{j\omega \epsilon_{0}}\sum_{n=-\infty }^{+\infty }a_{n}H_{n}^{(2)\prime }\left(k_{2}\rho \right)e^{jn\phi }, \end{array}$$

(38) $$\begin{array}{cc} E_{\phi }^{abs} & =-\frac{H_{0}k_{1}}{j\omega \epsilon_{1}}\sum_{n=-\infty }^{+\infty }b_{n}J_{n}^{\prime }\left(k_{1}\rho \right)e^{jn\phi }. \end{array}$$

In this section, we are solving for the TE mode of excitation, for which *n* varies from 0 to ∞. Let us use (35) to (38) to derive expressions for the internal and external admittance.

#### 3.2.1. Internal and External Admittance

In case of TE mode, with *n* varying from 0 to ∞, the internal admittance at the boundary of metallic nanowire will be reciprocal of $Z_{int}^{{TE}_{n}}$ under limits ($lim_{\rho \to a}$). Using the values of $H_{z}^{abs}$ (36) and $E_{\phi }^{abs}$ (38), $Y_{int}^{{TE}_{n}}$ can be written as

(39) $$Y_{int}^{{TE}_{n}}{|}_{lim_{\rho \to a}}=-\frac{1}{Z_{int}^{{TE}_{n}}{|}_{lim_{\rho \to a}}}=-\frac{H_{z}^{abs}}{E_{\phi }^{abs}}=\frac{j\omega \epsilon_{1}J_{n}\left(k_{1}a\right)}{k_{1}J_{n}^{\prime }\left(k_{1}a\right)},$$

where the negative sign is due to inward wave. Similarly, for the external admittance, using the values of $H_{z}^{sca}$ and $E_{\phi }^{sca}$ from (35) and (37), we can write

(40) $$Y_{ext}^{{TE}_{n}}{|}_{lim_{\rho \to a}}=\frac{1}{Z_{ext}^{{TE}_{n}}{|}_{lim_{\rho \to a}}}=-\frac{H_{z}^{sca}}{E_{\phi }^{sca}}=\frac{j\omega \epsilon_{2}H_{n}\left(k_{2}a\right)}{k_{2}H_{n}^{\prime }\left(k_{2}a\right)},$$

where $Y_{int}^{{TE}_{n}}$ (internal) and $Y_{ext}^{{TE}_{n}}$ (external) are the admittance of the excited *n*th TE mode.

#### 3.2.2. **TE** Mode: Cross-Sectional Areas Efficiencies Using Admittance

For the development of **TE** solution, we will follow the analogous approach used in solving **TM** case. The voltages are defined as follows:

- $V_{ext}^{TE}\left(k_{1}a\right)$: Voltage due to nanowire with property $k_{1}$, supporting scattering waves.
- $V_{ext}^{TE}\left(k_{2}a\right)$: Voltage due to nanowire with property $k_{2}$, supporting scattering waves.
- $V_{int}^{TE}\left(k_{1}a\right)$: Voltage due to nanowire with property $k_{1}$, supporting waves absorption.
- $V_{int}^{TE}\left(k_{2}a\right)$: Voltage due to nanowire with property $k_{2}$, supporting waves absorption.
- $Y_{\alpha }$: The ratio between $V_{int}^{TE}\left(k_{2}a\right)$ and $V_{ext}^{TE}\left(k_{2}a\right)$.

**Figure 6:**
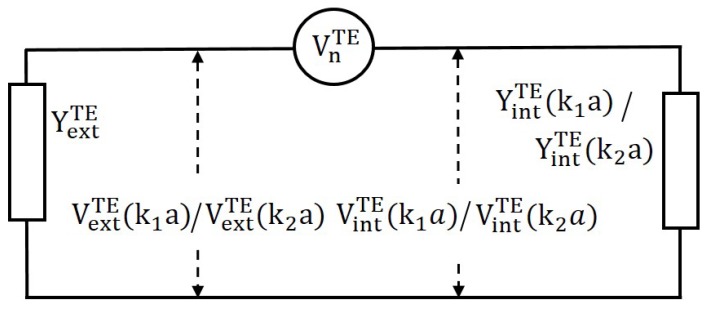
For the excitation of TE mode, a voltage generator $V_{n}^{TE}$ at the boundary of metallic nanowire, get distributed as internal ($V_{int}^{TE}\left(k_{1}a\right)$/$V_{int}^{TE}\left(k_{2}a\right)$) and external ($V_{ext}^{TE}\left(k_{1}a\right)$/$V_{ext}^{TE}\left(k_{2}a\right)$) voltage based on properties ($k_{1}$/$k_{2}$) of the internal medium as shown in Figure 4a,b. Whereas $Y_{int}^{n}\left(k_{1}a\right)$/$Y_{int}^{n}\left(k_{2}a\right)$ represents internal admittance of the nanocylinder with property $k_{1}$/$k_{2}$ and $Y_{ext}^{TM}\left(k_{2}a\right)$ is the external admittance of the nanocylinder.

The above voltages and their relationship are further shown in Figure 6. In TE mode, we define the scattering power due to excited mode as

(41) $$W_{s}^{TE}=2\pi aL\frac{{\left(V_{s}^{TE}\right)}^{2}}{2\eta_{2}}.$$

The 2πaL contribution is considering only the energy scattered per unit length by constructing an imaginary surface of length *L* and radius *a*. Please note that $V_{s}^{TE}$ is the voltage, which supports excitation of TE mode. It is defined using $a_{n}^{TE}$ (the scattering gain factor) and the incident wave generator ($V_{n}^{TE}$) as

(42) $$V_{s}^{TE}=V_{n}^{TE}a_{n}^{TE}.$$

Without loss of generality, a similar approach to Section 3.1.3 can be adopted and we can write value of $V_{ext}^{TE}\left(k_{1}a\right)$ and $V_{ext}^{TE}\left(k_{2}a\right)$ from Figure 6 as

(43) $$V_{ext}^{TE}\left(k_{1}a\right)=\frac{V_{n}^{TE}Y_{int}^{TE}\left(k_{1}a\right)}{Y_{int}^{TE}\left(k_{1}a\right)+Y_{ext}^{TE}\left(k_{2}a\right)},$$

(44) $$V_{ext}^{TE}\left(k_{2}a\right)=\frac{V_{n}^{TE}Y_{int}^{TE}\left(k_{2}a\right)}{Y_{int}^{TE}\left(k_{2}a\right)+Y_{ext}^{TE}\left(k_{2}a\right)}.$$

The gain factor $a_{n}^{TE}$ is defined as the fraction increase in voltage due to presence of the metallic cylindrical nanowire with material properties $k_{1}$, the value of $a_{n}^{TE}$ can be written as

(45) $$a_{n}^{TE}=1-\frac{V_{ext}^{TE}\left(k_{1}a\right)}{V_{ext}^{TE}\left(k_{2}a\right)}=\frac{V_{ext}^{TE}\left(k_{2}a\right)-V_{ext}^{TE}\left(k_{1}a\right)}{V_{ext}^{TE}\left(k_{2}a\right)}=\frac{\left[Y_{\alpha }Y_{int}^{TE}\left(k_{2}a\right)-Y_{int}^{TE}\left(k_{1}a\right)\right]}{\left[Y_{int}^{TE}\left(k_{1}a\right)+Y_{ext}^{TE}\left(k_{2}a\right)\right]}.$$

The gain defined by (45), exist due to the presence of metallic nanowire with property $k_{1}=\omega \sqrt{\epsilon_{1}\mu_{1}}$ to support TE mode. Using same step as in Section 3.1.3, we can prove solution for the TE mode for scattering cross-sectional area efficiency to have a general form, which can be written as

(46) $$Q_{sca}=\frac{W_{s}}{2aLP}=\frac{2}{x}\sum_{n=0}^{\infty }{\left|\frac{Y_{\alpha }\left[Y_{int}^{TE}\left(k_{1}a\right)-Y_{int}^{TE}\left(k_{2}a\right)\right]}{\left[Y_{int}^{TE}\left(k_{1}a\right)+Y_{ext}^{TE}\left(k_{2}a\right)\right]}\right|}^{2},$$

Using (39) and (40) in (46), the absolute term can be further reduced as

(47) $$\begin{array}{c} \left.\begin{array}{c} \frac{Y_{\alpha }\left[Y_{int}^{TE}\left(k_{1}a\right)-Y_{int}^{TE}\left(k_{2}a\right)\right]}{\left[Y_{int}^{TE}\left(k_{1}a\right)+Y_{ext}^{TE}\left(k_{2}a\right)\right]}=\\ \frac{mJ_{n}\left(k_{1}a\right)J_{n}^{\prime }\left(k_{2}a\right)-J_{n}^{\prime }\left(k_{1}a\right)J_{n}\left(k_{2}a\right)}{mJ_{n}\left(k_{1}a\right)H_{n}^{(2)\prime }\left(k_{2}a\right)-J_{n}^{\prime }\left(k_{1}a\right)H_{n}^{\left(2\right)}\left(k_{2}a\right)}=a_{n} \end{array}\right. \end{array}$$

Please note that from (4) and (9) $a_{n}=a_{n}^{TE}$. Therefore, the scattering cross-sectional area efficiency can be written as

(48) $$Q_{sca}=\frac{W_{s}^{TE}}{2aLI_{i}}=\frac{2}{x}\sum_{n=0}^{\infty }\left[|a_{0}{|}^{2}+2\sum_{n=1}^{+\infty }{\left|a_{n}\right|}^{2}\right].$$

Similarly, an expression can be derived for extinction and absorption cross-sectional area efficiencies for the ${\mathrm{TE}}_{n}$ mode using our proposed wave impedance model.

## 4. Simulation and Results

**Figure 7:**
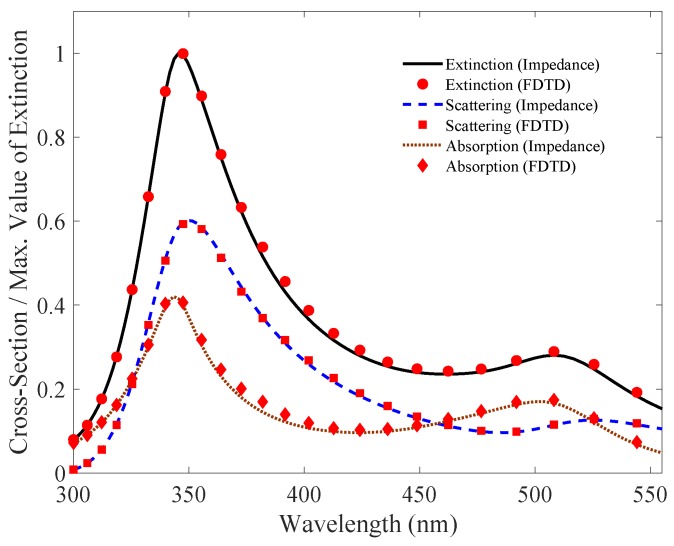
**TE** mode: Comparison of $Q_{ext}$, $Q_{sca}$ and $Q_{abs}$ simulation for a 40 nm radius cylindrical nanowire using our proposed impedance model with the full-wave field solver. The values are normalized to unity for the extinction cross-sectional area efficiencies, with scattering and absorption plot scaled accordingly. The simulation result show negligible difference between the two models.

**Figure 8:**
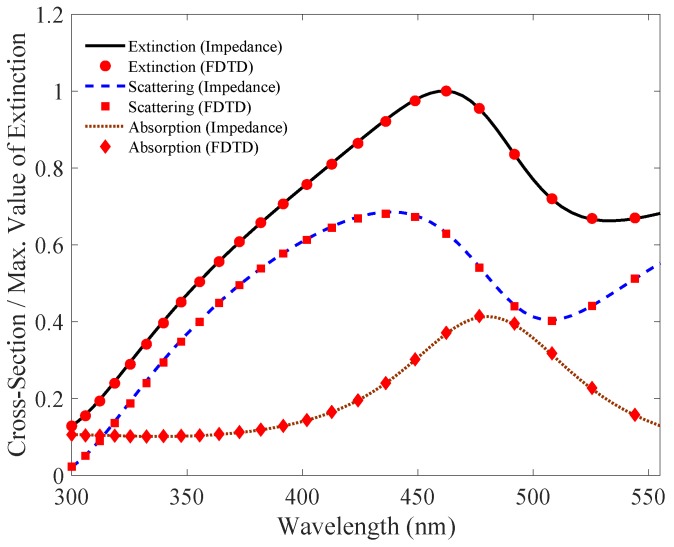
**TM** mode: Comparison of $Q_{ext}$, $Q_{sca}$ and $Q_{abs}$ simulation for a 40 nm radius cylindrical nanowire using our proposed impedance model with the full-wave field solver. The values are normalized to unity for the extinction cross-section efficiency, with scattering and absorption plot scaled accordingly. The simulation result show negligible difference between the two models.

In this section, we will analyze and simulate the proposed model. We will particularly focus on comparing our results with the simulation of full-wave field solvers. In this simulation, we will use gold cylindrical nanowire with permittivity represented by modified Drude dielectric function [30]. As derived in the preceding sections, the expressions for the impedances (internal and external) of the metallic nanowire are used to derive cross-sectional areas for **TE** and **TM** modes. In FDTD, the extinction, scattering and absorption cross-section efficiencies are solved using plane wave excitation and defining the separate regions of Total Field (TF) and Scattered Field (SF) for the measurement. The nanowire is placed in TF region, and the two regions are separated by virtual boundary. Absorbing conditions are used and the results of the cross-sectional efficiencies are plotted and compared with proposed impedance method in Figure 7 and Figure 8.

In Figure 7, we plot the normalized cross-sectional areas efficiencies for a 40 nm radius cylindrical nanowire using our proposed impedance model and compared it with the full-wave numerical field solver simulation. The solid black, blue and dotted red lines show extinction, scattering, and absorption cross-sectional areas efficiencies respectively for our proposed impedance model for the excited **TE** mode. The red dots show the results of field solver simulation. Please note that the simulation is for a 40 nm radius cylindrical nanowire, with normalized cross-sections values. In **TE** mode, the resonance peaks appear at 346 nm, 350 nm and 343.5 nm for the extinction, scattering, and absorption cross-sectional areas, respectively. Similarly, for the **TM** mode the results are plotted in Figure 8. The resonance peaks occur at 462 nm, 439 nm and 480.5 nm for the $Q_{ext}$, $Q_{sca}$ and $Q_{abs}$ respectively. The result shows negligible difference between the proposed impedance model and full-wave field solver simulation.

**Figure 9:**
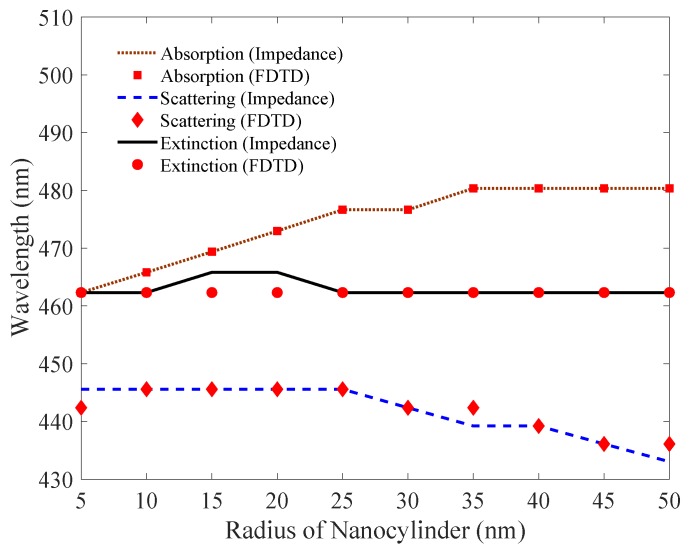
${\mathrm{TE}}_{n}$ mode extinction, scattering, and absorption cross-sectional areas efficiencies of cylindrical nanowire plotted with change in radius. The proposed model matches well with the full-wave field solution.

To complete the simulation, we will check the accuracy of our proposed model by plotting resonance frequencies and comparing it with the full-wave field solver solution. In Figure 9, we plot the extinction, scattering and absorption resonance frequency against change in the radius of the nanowire for the ${\mathrm{TE}}_{n}$ mode. In the plot, black solid line and brown dotted line shows our proposed impedance model and FDTD full-wave simulation, respectively. We note that a negligible difference is observed between our analytical close-form impedance model and the full-wave simulation. Metallic elements are metamaterials basic building blocks, and one of their interesting application is the rapidly evolving field of metamaterial sensing, where surface plasmon resonance can greatly enhance sensitivity of the sensor [31,32].

## 5. Conclusions

In this paper, we proposed an extended cylindrical wave impedance approach to model the metallic cylindrical nanowire at optical frequency. The motivation is the need to develop simplified models for scattering, absorption, and extinction efficiencies, which captures the main features of the original system. The wave impedance formulation works well at microwave for various mode of transmission. In this work we showed that for geometrically well-conditioned problem, a similar approach can be extended up to optical frequencies and the derived impedances can be used to adequately describe the behavior of metal at optical frequencies. The proposed method models a single metallic cylindrical nanowire, which once repeated periodically can achieve negative permittivity materials and is also the basic building block of many meta-devices and metamaterials. The impedance model provides the intuitive understanding of the qualitative behavior of the nanowire and facilitate the rapid, low-cost, and efficient future designs for the nanowire-based metamaterials.

## Abbreviations

The following abbreviations are used in this manuscript:

**E**	Electric
**M**	Magnetic
**TE**	Transverse Electric
**TM**	Transverse Magnetic
FDTD	Finite Difference Time Domain
